# Combined endovascular and surgical management of acute superior mesenteric artery embolism complicated by intestinal necrosis: a case report

**DOI:** 10.3389/fmed.2026.1796951

**Published:** 2026-03-20

**Authors:** Yanyan Liu, Gai Zhou, Jie Ying, Daoming Yan, Jianxin Ge, Zonghang Liu, Rongjia Zhang, Jianming Sun

**Affiliations:** 1 Department of Andrology, Seventh People’s Hospital affiliated to Shanghai University of Traditional Chinese Medicine, Shanghai, China; 2 Department of Gastroenterology, Affiliated Nanjing Jiangbei Hospital of Nantong University, Nanjing, China; 3 Interventional Department, Affiliated Nanjing Jiangbei Hospital of Nantong University, Nanjing, China; 4 Department of General Surgery, Affiliated Nanjing Jiangbei Hospital of Nantong University, Nanjing, China; 5 Department of Immunology, Medical School of Nantong University, Nantong, China

**Keywords:** acute superior mesenteric artery embolism, arteriography, CTA of the abdomen, intestinal necrosis, multidisciplinary joint intervention

## Abstract

**Background:**

Acute superior mesenteric artery embolism (ASMAE) is a clinically rare acute abdominal condition and a type of acute mesenteric ischemia. It is characterized by sudden onset, rapid progression, and a high rate of misdiagnosis. Due to atypical early symptoms (such as dissociation between abdominal pain and physical signs), diagnosis and treatment are often delayed. Currently, there is no highly specific biomarker available for definitive diagnosis. Treatment typically involves anticoagulation and vasospasmolysis, with interventional or surgical intervention selected based on the extent of intestinal wall necrosis.

**Case:**

For a 42-year-old male patient with superior mesenteric artery embolism complicated by intestinal necrosis, continuous monitoring was carried out through interventional therapy, pharmacological support, and surgical treatment, along with abdominal CTA, arteriography, CT scans, and clinical examinations. Follow-up and timely re-examinations were conducted after discharge.

**Results:**

The patient was successfully treated with a sequential therapeutic approach combining “interventional thrombectomy/thrombolysis” and “surgical bowel resection.” Interventional treatment partially recanalized the occluded vessel but failed to completely prevent intestinal necrosis. Timely surgical intervention removed 10 cm of necrotic small intestine, preventing further deterioration of the condition. Postoperative vascular imaging showed significant improvement in superior mesenteric artery blood flow. The patient eventually recovered and was discharged, with recent follow-up showing no discomfort.

**Conclusion:**

The clinical manifestations and signs of this case of superior mesenteric artery embolism were atypical, and no abnormalities were found in D-dimer and lactate upon admission. However, when intestinal necrosis occurs, the early laboratory findings and signs become more typical. Through timely CT and CTA imaging assessments, dynamic monitoring of the condition, and multidisciplinary intervention, successful treatment was achieved, providing valuable insights for the diagnosis and management of similar cases.

## 1 Background

Acute mesenteric ischemia refers to a sudden interruption of blood flow to the small intestine and includes four main entities: superior mesenteric artery embolism, superior mesenteric artery thrombosis, mesenteric venous thrombosis, and non-occlusive mesenteric ischemia (1–4). Acute superior mesenteric artery embolism (ASMAE) is relatively rare and is often characterized by a marked dissociation between symptoms and physical signs, which makes it prone to missed or delayed diagnosis during clinical practice (5, 6). Atrial fibrillation, myocardial ischemia, valvular heart disease, and other conditions associated with atrial thrombus formation are the principal risk factors for acute mesenteric embolism.

To date, no highly specific biomarker is available for the definitive diagnosis of ASMAE (7, 8). Serum lactate and D-dimer are currently regarded as relatively sensitive indicators in thromboembolic diseases. However, novel biomarkers such as intestinal fatty acid–binding protein and citrulline lack sufficient sensitivity and specificity (9). Common imaging modalities, including computed tomography angiography (CTA) and digital subtraction angiography (DSA), are essential for diagnosis. Nevertheless, some patients decline these examinations at an early stage because of cost considerations, resulting in delayed diagnosis and treatment (10). When intestinal necrosis occurs, patients typically present with colicky abdominal pain accompanied by diarrhea, abdominal distension, and hematochezia (11, 12). Pain is usually refractory to antispasmodic therapy, and signs of peritoneal irritation may be evident on physical examination. Once ASMAE is diagnosed, prompt initiation of fluid resuscitation, anticoagulation, and relief of mesenteric vasospasm is required, followed by endovascular or surgical intervention based on the extent of bowel necrosis.

## 2 Case presentation

A 42-year-old man was admitted on 8 December 2024, at 14:14 with a 4-h history of abdominal pain accompanied by nausea, vomiting, and diarrhea. Four hours prior to admission, he developed paroxysmal cramping pain in the epigastric and periumbilical regions without an obvious trigger, associated with nausea and 3–4 episodes of non-projectile vomiting of gastric contents. He also reported three episodes of loose yellow stools. There was no chest pain, dyspnea, dizziness, fever, hematochezia, or melena.

Non-contrast abdominal CT performed in the emergency department showed thickening and roughening of the walls of portions of the small bowel and the colon and rectum, with a small amount of intraluminal fluid in some small-bowel loops. Laboratory tests revealed a white blood cell count of 10.5 × 10^9^/L and a neutrophil count of 6.65 × 10^9^/L. Liver function tests, pancreatic enzymes, electrolytes, creatine kinase, creatinine, alanine aminotransferase, amylase, D-dimer, cardiac troponin I, and electrocardiography were unremarkable. After analgesic treatment, his abdominal pain improved, and he was admitted to the gastroenterology department with a provisional diagnosis of abdominal pain.

His medical history was notable for lower limb arterial occlusive disease treated with balloon angioplasty at another hospital in January 2024. He had been taking aspirin and clopidogrel long term but discontinued both medications one month prior to admission. Physical examination on admission revealed mild diffuse abdominal tenderness without other positive findings.

After admission, the patient was managed with fasting, intravenous fluids, empirical antibiotic therapy with levofloxacin, acid suppression with esomeprazole, and antispasmodic treatment. Although his abdominal pain temporarily improved, he experienced recurrent episodes. Repeat laboratory tests 16 h after admission showed no significant progression. CTA was recommended to exclude vascular pathology, but the patient initially declined because of financial concerns. An urgent D-dimer test remained within normal limits.

**FIGURE 1:**
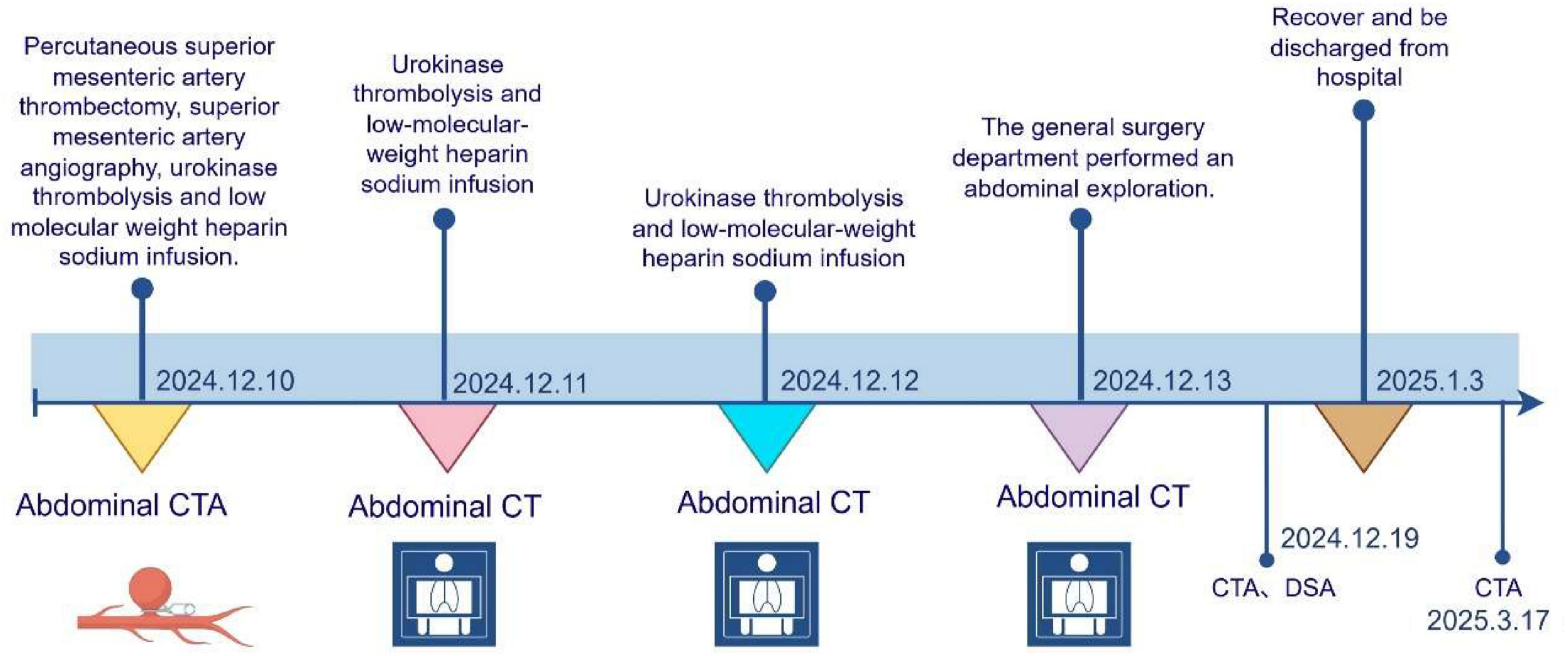
The timeline figure. 10 December 2024: The patient is experiencing abdominal distension and discomfort. The abdominal CTA scan revealed embolism of the superior mesenteric artery. Performed “percutaneous embolization of the superior mesenteric artery and superior mesenteric artery angiography,” followed by the infusion of 300,000 U of urokinase for thrombolytic treatment, and then continuous infusion of low molecular weight heparin sodium. 11 December 2024: The patient is experiencing abdominal distension and discomfort. The abdominal CT scan showed mild dilation and fluid accumulation in some parts of the small intestine. 500,000 units of urokinase were used for thrombolysis, and low molecular weight heparin sodium was continuously infused after the thrombolysis. 12 December 2024: The patient complained of abdominal pain and felt intestinal peristalsis. On physical examination, the abdomen was soft, with tenderness but no rebound tenderness or muscle tension. The abdominal CT scan showed that part of the small intestine was significantly dilated with fluid accumulation and multiple fluid levels. 500,000 units of urokinase were used for thrombolysis, and low molecular weight heparin sodium was continuously infused after the thrombolysis. 13 December 2024: The patient complained of severe abdominal distension, without obvious abdominal pain. Physical examination: The abdomen was significantly distended, soft, with tenderness, rebound tenderness, and no muscle tension. The abdominal CT scan revealed dilation and accumulation of gas and fluid in the left half of the small intestine, along with surrounding exudation. A small amount of blood was also found in the abdominal and pelvic cavities. Considering the clinical manifestations, it is highly likely that there is a blood supply-related intestinal obstruction with possible intestinal necrosis. General surgery department performed an abdominal exploration. 19 December 2024: Abdominal CTA, DSA. 3 January 2025: Recover and be discharged from hospital. 17 March 2025: Abdominal CTA.

Forty-two hours after admission, the patient consented to CTA, which demonstrated embolism of the superior mesenteric artery. Interventional radiology consultation suggested embolism of the superior mesenteric artery, and endovascular thrombectomy and thrombolysis were recommended to prevent intestinal ischemia and necrosis. With informed consent, percutaneous superior mesenteric artery thrombectomy and angiography were performed on 10 December 2024, at 12:15 (46 h after admission) (Figure 1).

During the procedure, the patient was placed in the supine position under standard sterile conditions. The right femoral artery was selected as the puncture site, and after successful local anesthesia with 2% lidocaine, a 5F vascular sheath was inserted using the Seldinger technique. Under the guidance of a hydrophilic guidewire, a 5F RH catheter was advanced to the origin of the superior mesenteric artery. Angiography demonstrated filling defects in the mid-to-distal segments of the superior mesenteric artery, with minimal visualization of distal small branches, suggesting superior mesenteric artery embolism caused by thrombus. Because further catheter advancement was difficult, a 260-cm guidewire was introduced, and the sheath was exchanged for a 6F disposable kink-resistant sheath. A 5F Cobra catheter was advanced into the proximal superior mesenteric artery; however, the occluded segment could not be traversed. A microguidewire and microcatheter were then introduced in an attempt to cross the occlusion. Intra-arterial thrombolysis with 100,000 units of urokinase was subsequently administered. After exchanging for a 6F guiding catheter, a small amount of thrombus was aspirated, but thrombectomy remained difficult. The catheter was then replaced with a 6F support catheter, which was advanced into the occluded segment, and a large amount of thrombus was successfully aspirated under negative pressure.

Repeat angiography demonstrated visualization of the mid-to-distal segments of the superior mesenteric artery and some distal branches, with markedly improved blood flow compared with before; however, filling defects in some small arteries were still observed. An additional 100,000 units of urokinase were administered for thrombolysis. The 6F kink-resistant sheath was retained, and a 4F thrombolysis catheter was introduced into the main trunk of the superior mesenteric artery for continuous catheter-directed thrombolysis, with the catheter sealed using a heparin cap. A total of 2,000 units of heparin were administered intraoperatively. During the procedure, the patient’s abdominal pain was slightly relieved, and the sheath was secured with sterile dressing, with compression bandaging applied at the puncture site. The patient was then transferred back to the ward in stable condition. The patient was instructed to maintain strict lower-limb immobilization and bed rest and was treated with anticoagulation, thrombolysis, analgesia, and other supportive therapies, with continued observation and symptomatic management.

Postoperatively, repeat laboratory tests, including complete blood count, emergency biochemistry, cardiac troponin T, coagulation profile, and arterial blood gas analysis, showed no significant progression. Catheter-directed thrombolysis with urokinase (300,000 units diluted in 48 mL of 0.9% normal saline at a rate of 16 mL/h) was initiated. After completion of thrombolysis, continuous infusion of low-molecular-weight heparin sodium (1,000 units diluted in 50 mL of 0.9% normal saline at a rate of 4 mL/h) was administered, along with long-term subcutaneous low-molecular-weight heparin sodium at a dose of 5,000 units every 12 h.

On postoperative day 1 (December 11), repeat complete blood count and renal function tests showed no significant progression. The patient complained of abdominal distension and a discomfort, and repeat whole-abdominal CT showed no obvious changes. Thrombolytic therapy with an additional 500,000 units of urokinase was administered, followed by continuous infusion of low-molecular-weight heparin sodium. DSA performed 24 h postoperatively demonstrated partial visualization of distal branches of the superior mesenteric artery, with significantly improved blood flow compared with prior imaging, although minor filling defects in some small arteries persisted. Antimicrobial therapy was adjusted to cefoperazone–sulbactam sodium at 3 g every 12 h in combination with levofloxacin.

On postoperative day 2 (December 12), the patient reported subjective abdominal pain and a sensation of bowel peristalsis. Physical examination revealed a soft abdomen with tenderness but without rebound tenderness or muscle guarding. Repeat laboratory tests, including complete blood count, renal function, and coagulation profile, showed no significant progression. Thrombolysis with 500,000 units of urokinase was continued, followed by continuous infusion of low-molecular-weight heparin sodium. Repeat abdominal CT showed no progression.

On postoperative day 3 (December 13), the patient complained of marked abdominal distension without significant abdominal pain. Physical examination revealed obvious abdominal distension with a soft abdomen, accompanied by tenderness and rebound tenderness, but without muscle guarding. Gastrointestinal decompression was initiated. An urgent abdominal CT scan demonstrated dilation of the left-sided small bowel with gas and fluid accumulation and surrounding exudation, along with a small amount of hemoperitoneum in the abdominopelvic cavity. Based on the imaging findings in combination with the clinical presentation, ischemic bowel obstruction complicated by intestinal necrosis was considered highly likely, and close clinical correlation and follow-up were recommended. An emergency consultation with the general surgery department was obtained, and transfer to the surgical service for further operative management was recommended. With informed consent obtained from the patient and his family, the patient was transferred to the Department of General Surgery at 12:27 on December 13 for further treatment, and the exploratory laparoscopy was performed at 13:38.

Surgical procedure: After successful induction of anesthesia, the patient was placed in the lithotomy position, and routine skin preparation and sterile draping were performed. A transumbilical puncture was made to establish pneumoperitoneum with an intra-abdominal pressure of 15 mmHg. Reusable 5-mm trocars were inserted into the left upper and lower quadrants, and laparoscopic instruments were introduced. Exploration of the abdominal cavity revealed approximately 500 mL of bloody ascites. The intestinal loops were markedly edematous and dilated. The greater omentum was found to be wrapped around the proximal jejunum. After separation, a segment of jejunum located approximately 60–70 cm distal to the ligament of Treitz appeared black in color, with a firm consistency and a purulent coating on the surface. The mesentery showed scattered congestion and edema, and no additional areas of bowel necrosis were identified. A red urinary catheter was placed through the mesentery of the necrotic bowel segment for traction. A 15F silicone drain was placed in the pelvic cavity. The pneumoperitoneum was released, and the laparoscopic instruments were removed. A 5-cm left transrectus abdominal incision was made, and the abdominal wall was entered layer by layer with protection of the incision. The small intestine was exteriorized through the incision, and a stoma was fashioned with appropriate tension. The proximal and distal segments of the small intestine were transected using an 80 L linear cutting stapler, and a total of 10 cm of small intestine was resected. The proximal and distal bowel ends were sutured and fixed, and the peritoneum and fascia were secured to the serosa of the small intestine and opened. A stoma appliance was applied. The procedure was completed successfully, with an estimated intraoperative blood loss of 50 mL. Intraoperative vital signs remained stable, and the patient was transferred back to the ward in stable condition after surgery. Postoperatively, the patient received antimicrobial therapy, acid suppression, and nutritional support. Enoxaparin sodium injection at a dose of 4,000 IU was administered subcutaneously every 12 h.

On postoperative day 3, repeat DSA performed on 16 December 2024, demonstrated visualization of most branches of the superior mesenteric artery, with minor filling defects in some small arteries. The marginal arterial collaterals were well developed, the capillary network was rich, and blood flow was markedly improved compared with previous imaging.

On postoperative day 6 (19 December 2024), repeat abdominal computed tomography angiography (CTA) demonstrated a small amount of mural thrombus in the proximal-to-mid segment of the superior mesenteric artery. Compared with the previous imaging performed on 10 December 2024, the thrombus burden was markedly reduced. Nutritional support therapy was continued, and the patient tolerated advancement to an oral diet without any discomfort. The patient subsequently improved and was discharged on 13 January 2025. After discharge, the patient was instructed to take rivaroxaban 15 mg orally twice daily for the first three weeks, with the tablets crushed into powder and taken with meals. From the fourth week, the anticoagulation regimen was adjusted to rivaroxaban 10 mg once daily combined with clopidogrel 75 mg once daily. Three months after surgery, the patient was readmitted on 17 March 2025, for follow-up abdominal CTA. Imaging demonstrated postoperative changes consistent with jejunostomy, as well as calcified and non-calcified plaques in the distal abdominal aorta, resulting in mild luminal stenosis. On 25 March 2025, the patient underwent jejunostomy closure combined with laparoscopic adhesiolysis. The postoperative course was uneventful, and the patient was discharged in improved condition on 3 April 2025. After discharge, clopidogrel and rivaroxaban were continued. Recent telephone follow-up revealed no discomfort or adverse symptoms.

The rationale for intermittent administration of urokinase in catheter-directed thrombolysis includes: (1) increasing local drug concentration to enhance thrombolytic efficiency; (2) reducing systemic drug exposure and bleeding risk; and (3) mechanical adjunctive effects and hemodynamic considerations. Considerations: Firstly, patient factors: (1) Time since embolization and intestinal viability: typically suitable for patients with a short symptom duration (generally < 12 h) and no evidence of transmural intestinal necrosis. (2) Underlying diseases and bleeding risk. Secondly, technical factors: (1) Catheter tip position: The catheter must be precisely placed within the thrombus or just proximal to it to ensure effective local drug delivery. (2) Combination therapy: Often performed alongside mechanical thrombectomy or aspiration. Thirdly, monitoring and evaluation: Angiographic monitoring, clinical symptom assessment, laboratory monitoring, and organ function monitoring.

Clinical reasoning for continued urokinase use after DSA: DSA revealed partial opacification of the distal branches of the SMA. Considering the recanalization of the main trunk, but with the presence of a significant number of small thrombi in the distal secondary branches or microcirculation, under the premise of clinically ruling out intestinal necrosis and bleeding risk, systemic fibrinolysis and anticoagulation were employed in an attempt to dissolve distal microthrombi inaccessible to the catheter and inhibit new thrombus formation. The goal was to further improve blood perfusion in the mesenteric artery within a limited time window, thereby avoiding secondary surgery or extensive bowel resection. The subsequent intra-abdominal hemorrhage detected in the abdominal and pelvic cavity was a manifestation of intestinal necrosis, associated with intestinal ischemia and unrelated to the use of urokinase.

## 3 Discussion

During the course of the disease, the patient’s follow-up chest CTA revealed a possible mural thrombus in the thoracic aorta, which may be a risk factor for embolism or a risk factor for thrombosis. However, in the patient’s abdominal CTA findings, the thrombus was located in the middle and distal segments beyond the vascular opening. The vessel itself appeared smooth with no significant calcified plaques, which is consistent with the imaging characteristics of an embolism. Clinically, thrombosis is often preceded by prodromal symptoms of chronic intestinal ischemia (such as postprandial abdominal pain and weight loss), with symptoms worsening sharply after complete occlusion. In this case, the patient experienced an acute onset with no prodromal symptoms, which aligns with the clinical presentation of an embolism. ASMAE is an uncommon but life-threatening condition that is frequently misdiagnosed because of its nonspecific presentation and the dissociation between symptoms and physical findings. Disease progression is often rapid, and delayed treatment may result in transmural intestinal necrosis with a reported mortality rate of 60%–100% (13–15).

In the early stage of acute intestinal ischemia, ischemic injury is limited to the mucosal and submucosal layers of the intestine. Patients predominantly present with nonspecific gastrointestinal discomfort, most commonly characterized by sudden-onset, cramping pain in the periumbilical or epigastric region, often accompanied by diarrhea, abdominal pain, and hematochezia. On physical examination, abdominal tenderness is usually mild, the abdominal wall is soft without muscular guarding, and bowel sounds are normal or even hyperactive. Notably, abdominal pain is often refractory to antispasmodic therapy. In the present case, the patient initially manifested only paroxysmal, cramping abdominal pain, which was relieved by antispasmodic treatment. Further history taking revealed alcohol consumption prior to symptom onset, which easily led to misdiagnosis as acute gastroenteritis. In the intermediate stage of acute intestinal ischemia, ischemic injury extends further to involve the serosal layer. At this stage, due to reduced sensitivity of nociceptors within the intestinal wall, a so-called “pseudo-painless period” may occur approximately 3–6 h after the onset of abdominal pain. In the late stage of acute intestinal ischemia, the bowel becomes congested, and bloody exudative fluid accumulates within the abdominal cavity. Patients frequently develop hematochezia or hematemesis, and physical examination typically reveals overt signs of peritoneal irritation, with bowel sounds becoming diminished or even absent. In severe cases, bacterial translocation from the intestinal lumen may occur, resulting in severe peritonitis, secondary sepsis, and septic shock. In this case, when intestinal necrosis developed, the patient presented primarily with abdominal distension, without significant abdominal pain, hematemesis, or hematochezia, although signs of peritoneal irritation were evident on physical examination. The common cause of mesenteric artery stenosis or occlusion is atherosclerosis, so dual antiplatelet therapy is usually recommended after surgery, followed by long-term maintenance with a single drug (such as aspirin) to reduce the risk of in-stent thrombosis. Discontinuation of dual antiplatelet therapy is an important potential factor for disease recurrence in these patients.

**FIGURE 2:**
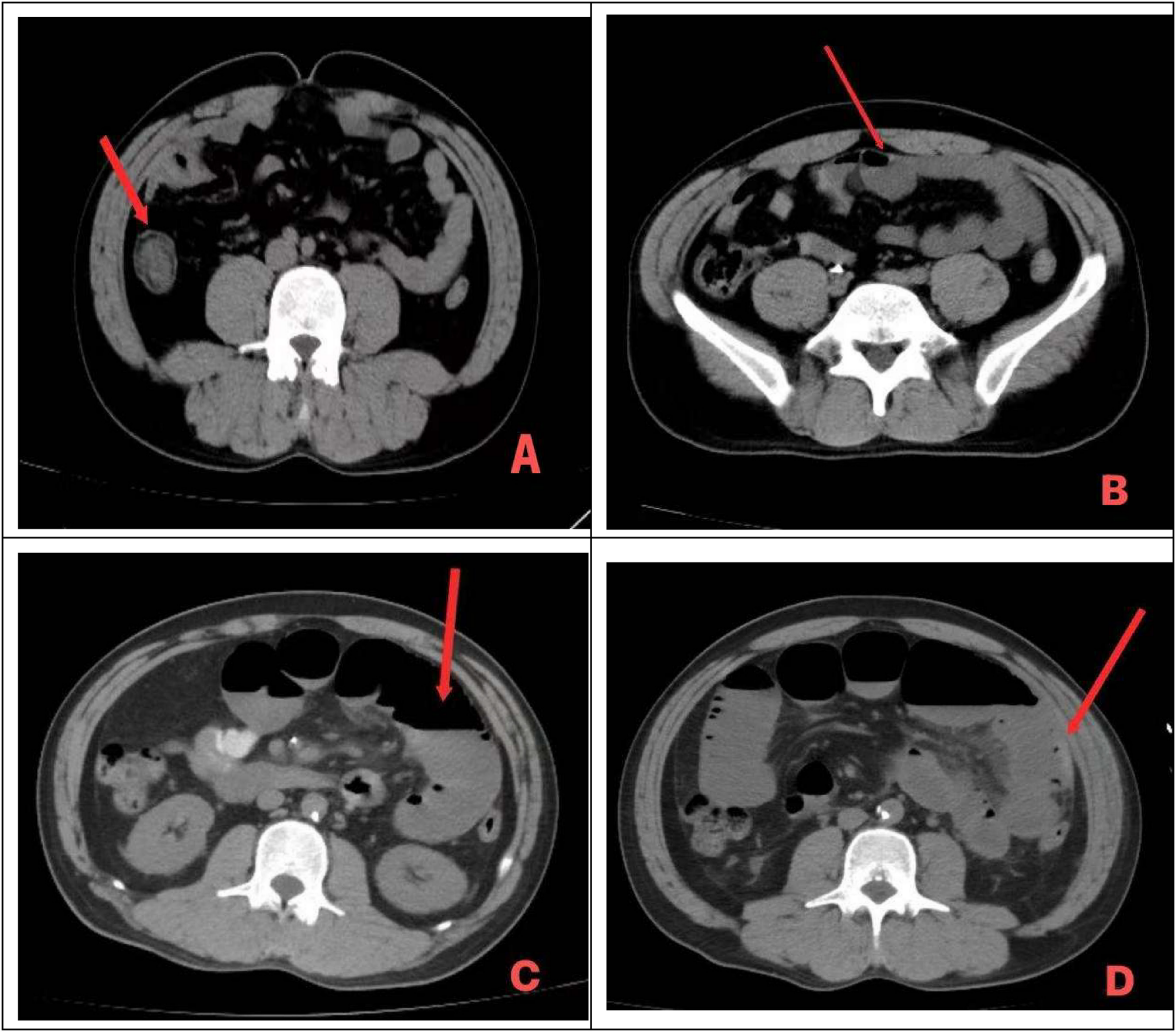
Non-contrast abdominal CT findings of the patient. **(A)** Abdominal CT on admission (8 December 2024) showing thickened and irregular bowel walls. **(B)** Follow-up CT on postoperative day 1 after endovascular intervention (11 December 2024) demonstrating mild dilation of some small bowel loops with fluid accumulation. **(C)** Follow-up CT on postoperative day 2 after endovascular intervention (12 December 2024) showing marked dilation of some small bowel loops with fluid accumulation and multiple air–fluid levels. **(D)** Follow-up CT on postoperative day 3 after endovascular intervention (13 December 2024) demonstrating dilation of the left-sided small bowel with gas and fluid accumulation, accompanied by surrounding exudation and a small amount of hemoperitoneum in the abdominopelvic cavity.

At present, no highly specific biomarkers are available for the definitive diagnosis of superior mesenteric artery embolism. Previous studies have shown that several laboratory parameters, including blood lactate levels, inflammatory markers such as C-reactive protein, and hematological indicators such as lactate dehydrogenase, have some value in predicting ASMAE (16–18). However, their sensitivity and specificity remain limited (19). Among these markers, D-dimer is currently recognized as a relatively sensitive indicator in thromboembolic diseases. However, in the present case, plasma D-dimer levels remained within the normal range both in the early stage of disease onset and at the time of concomitant intestinal necrosis (20–22). Therefore, the sensitivity of D-dimer in predicting intestinal ischemia and necrosis requires further investigation, and the findings reported by Block et al. (23) are consistent with the results observed in this case. Recent studies have suggested that multidetector computed tomography (MDCT) plays an important role in the diagnosis and prognostic evaluation of superior mesenteric artery embolism (24, 25). Nevertheless, this technique has not yet been widely implemented in routine clinical practice. As shown in Figure 2, when intestinal necrosis develops, imaging typically demonstrates progressive bowel dilatation, intraluminal fluid accumulation, and perienteric exudation. This case also underscores the importance of abdominal CTA in patients with persistent, refractory abdominal pain. Early completion of CTA to exclude ischemic bowel disease may help prevent diagnostic delay and improve patient prognosis (26–29).

**FIGURE 3:**
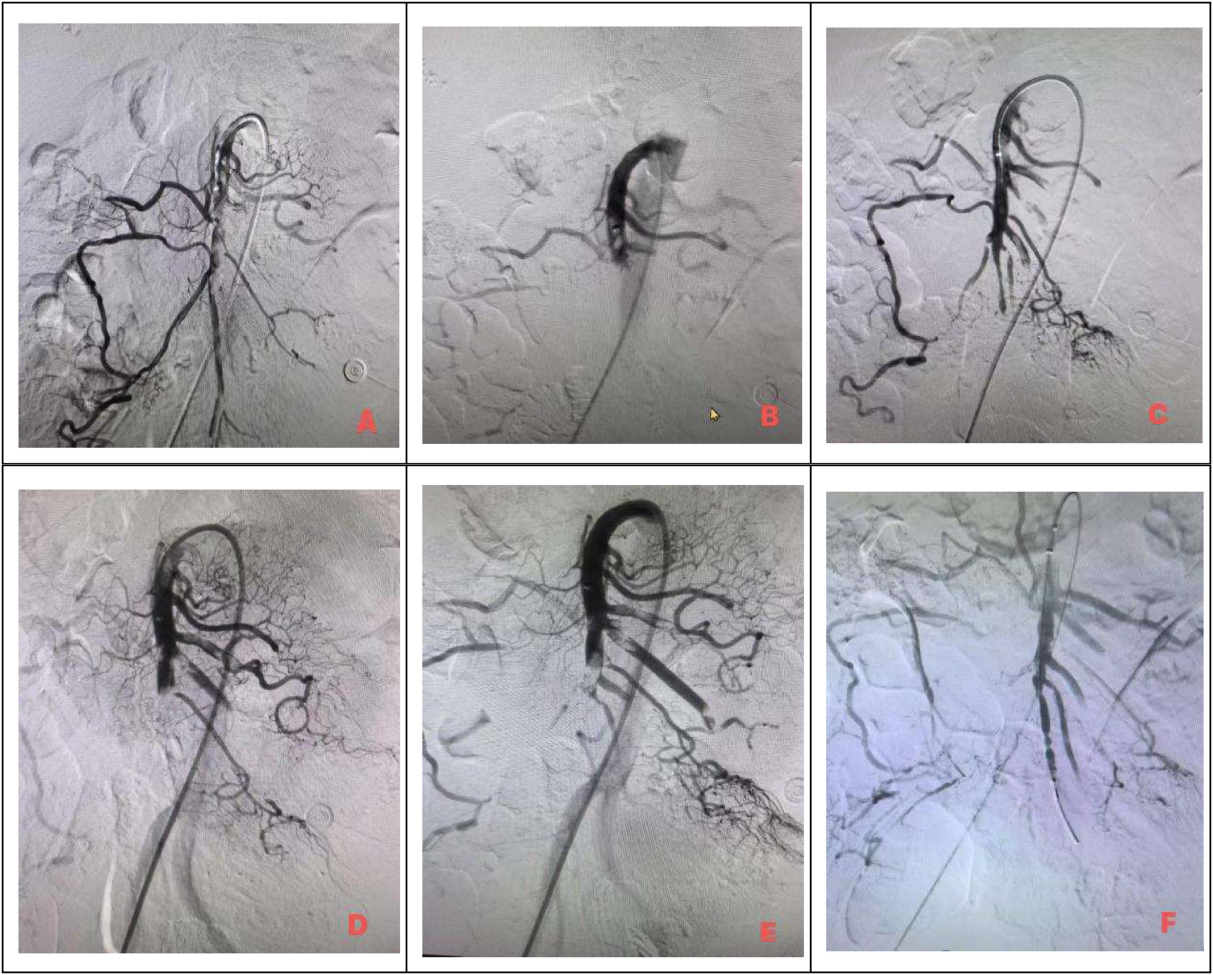
Digital subtraction angiography (DSA) images before and after endovascular treatment. **(A–C)** DSA performed on 10 December 2024, demonstrating filling defects in the mid-to-distal segments of the superior mesenteric artery, with limited visualization of distal small vessels; repeat angiography after catheter-directed thrombolysis and thrombectomy showed visualization of the mid-to-distal superior mesenteric artery and some distal branches, with markedly improved blood flow compared with baseline. **(D–F)** Follow-up DSA performed on 16 December 2024, after surgical intervention demonstrated visualization of most branch arteries of the superior mesenteric artery, with minor filling defects in some small arteries; the marginal arterial collaterals and capillary network were well developed, and blood flow was markedly improved compared with previous imaging.

**FIGURE 4:**
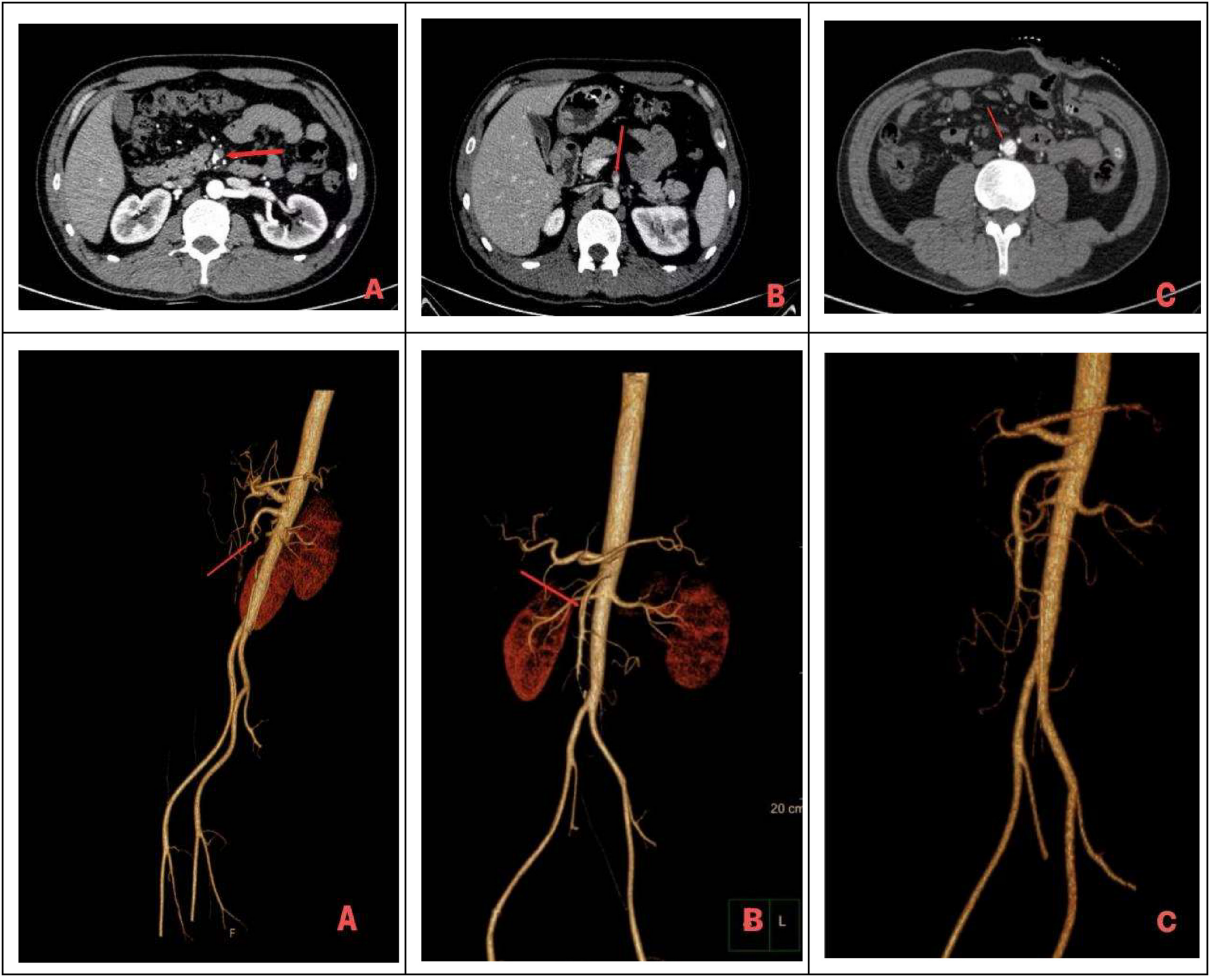
Changes in computed tomography angiography (CTA) and volume rendering (VR) images before and after endovascular and surgical interventions. **(A)** CTA and VR images obtained on 10 December 2024, before endovascular intervention, demonstrating superior mesenteric artery embolism. **(B)** Follow-up CTA and VR images obtained on 19 December 2025, after surgical intervention, showing a small amount of mural thrombus in the mid-segment of the superior mesenteric artery, with a marked reduction in thrombus burden compared with panel **(A)**. **(C)** Follow-up CTA and VR images obtained three months postoperatively (17 March 2025), demonstrating a smooth superior mesenteric artery wall without significant stenosis or focal dilation.

Clinical management of superior mesenteric artery embolism mainly includes supportive care, endovascular therapy, and surgical intervention. Rapid restoration of mesenteric blood flow is the key determinant of prognosis (30, 31). A retrospective study including 32 patients with superior mesenteric artery embolism reported that among 18 patients who underwent endovascular therapy, 12 successfully avoided open surgery, and the overall 30-day mortality rate was lower than that of patients treated with open surgery (16.7% vs. 33.3%) (32). At present, endovascular therapy is increasingly regarded as an alternative to open surgery, which represents a consensus across multiple studies. Current endovascular treatment modalities mainly include catheter-directed thrombolysis, mechanical thrombectomy (such as the Rotarex, AngioJet, and Penumbra systems), percutaneous transluminal angioplasty, stent implantation, and combinations of these strategies. However, thrombolytic therapy has several limitations, including the need for large doses of urokinase, an increased risk of bleeding, and prolonged treatment duration; therefore, coagulation function must be closely monitored during therapy. Once signs of peritonitis or clinical deterioration occur, immediate surgical intervention is indicated. The current trend favors a combined approach integrating open surgery with endovascular intervention to achieve better therapeutic outcomes. Studies have shown that endovascular therapy can be used as a preparatory measure before open surgery, helping to improve the patient’s general condition and reduce the risk of postoperative complications. In the present case, the patient underwent endovascular thrombolysis and thrombectomy, followed by postoperative urokinase thrombolysis and continuous anticoagulation with low-molecular-weight heparin sodium. As shown in Figures 3, 4, postoperative abdominal CTA demonstrated a marked reduction in thrombus burden and significantly improved patency of the superior mesenteric artery branches, although some vessels remained occluded. During treatment, coagulation function was monitored twice daily, and thrombolytic dosages were adjusted accordingly, and no bleeding events occurred throughout the entire treatment course.

When patients develop peritonitis or even intestinal necrosis, or when a single treatment modality yields unsatisfactory results, a combined approach integrating endovascular therapy and surgical intervention has been increasingly recognized by clinicians. A prospective study identified the following predictors of intestinal necrosis: (1) organ failure; (2) serum lactate levels ≥ 2 mmol/L; and (3) small bowel dilation with a diameter ≥ 2.5 cm (33). Conservative management is recommended when none of these factors are present, whereas immediate surgical intervention is advised when all three factors are present. When one or two factors are present, early surgical treatment is recommended in patients with elevated serum lactate levels (34, 35). However, in the present case, none of the above three factors were present. Before the development of intestinal necrosis, the patient’s complete blood count and inflammatory markers showed no significant abnormalities, and small bowel dilation did not reach 2.5 cm, which was inconsistent with the findings of the aforementioned study. On the third day after endovascular intervention, the patient presented only with abdominal distension, abdominal fullness, and localized signs of peritonitis. Meanwhile, abdominal CT demonstrated intramural gas accumulation and bowel lumen dilation, strongly suggesting the possibility of intestinal necrosis. Therefore, surgical intervention was promptly performed. The procedure was completed successfully, with a total of 10 cm of necrotic small bowel resected, and the patient was discharged in improved condition 16 days after surgery. In this case, the predictive value of hematological parameters for intestinal necrosis warrants further investigation. Short-interval postoperative CT re-examination and meticulous physical examination after endovascular intervention play a critical role in the early diagnosis of intestinal necrosis and in improving patient outcomes.

In 2025, Wang and Du (15) reported a case report, a patient was admitted because of acute abdominal pain, and CTA examination showed a filling defect in the SMA. The patient was diagnosed with ASMAE and underwent emergency surgical treatment for a thrombectomy and thrombolysis of the SMA under general anesthesia. After surgery, recanalization of the SMA was achieved. The patient was followed up six months later, and SMA CTA revealed that the main trunk and branches of the SMA were well visualized. They found CTA could provide a diagnosis with high sensitivity and specificity for ASMAE. In 2023, Simone Bongiovanni et al. (36) reported a case report that ultrasound assisted catheter directed thrombolysis of an embolic partial occlusion of the SMA. The treatment strategy resulted in complete dissolution of the multiple emboli and improved blood flow into the intestinal wall. After treatment, the patient’s condition has improved significantly. Also in 2023, Jing et al. (37) reported a case report that ASMAE was initially misdiagnosed as mechanical intestinal obstruction. After a detailed examination, a diagnosis of ASMAE was confirmed. They did not elaborate on how to carry out the treatment, but the case report emphasized that targeted examinations can lead to a timely diagnosis of ASMAE. In comparison to the three case reports mentioned above, this case of SMA embolism presented with atypical symptoms and signs. However, upon admission, D-dimer and lactate levels were found to be within normal ranges. When intestinal necrosis occurs, the preliminary laboratory findings and clinical signs tend to be more typical. This case report aims to provide a reference for clinicians in their diagnosis and treatment practices.

In summary, patients with ASMAE often present with nonspecific clinical manifestations and lack characteristic laboratory abnormalities, and even when intestinal necrosis develops, both symptoms and laboratory findings may remain nonspecific. As a result, this condition is highly prone to misdiagnosis, treatment is frequently delayed, and intestinal necrosis following endovascular therapy may not be recognized in a timely manner. Under such circumstances, early completion of abdominal CTA, repeated physical examinations, and short-interval follow-up CT imaging are crucial for accurate diagnosis and appropriate management. Meanwhile, there is an urgent need for highly specific and sensitive biomarkers to predict disease onset and progression in clinical practice, and the predictive value of indicators such as plasma D-dimer and lactate requires further investigation.

## 4 Conclusion

Superior mesenteric artery embolism complicated by intestinal necrosis is a critical condition. Early diagnosis requires combining abdominal CTA, short-term follow-up CT scans, and repeated physical examinations. In clinical diagnosis and treatment, there is an urgent need for highly specific and sensitive indicators to predict the occurrence and progression of the disease, while the predictive value of blood markers such as D-dimer and lactic acid requires further study. In terms of treatment, a personalized “interventional-surgical combined” approach can be adopted. Multidisciplinary collaboration and comprehensive postoperative supportive care can significantly improve prognosis.

## Data Availability

The original contributions presented in this study are included in this article/supplementary material, further inquiries can be directed to the corresponding authors.
